# Precise Confinement and Position Distribution of Atomic Cu and Zn in ZSM-5 for CO_2_ Hydrogenation to Methanol

**DOI:** 10.3390/nano13233053

**Published:** 2023-11-29

**Authors:** Hongxin Ding, Jinwen Zhang, Wenhua Feng, Qingying Yao, Li Zhang, Yuanhang Ren, Lin Ye, Bin Yue, Heyong He

**Affiliations:** Shanghai Key Laboratory of Molecular Catalysis and Innovative Materials, Department of Chemistry, Fudan University, Shanghai 200438, China; 19110220065@fudan.edu.cn (H.D.); 19110220077@fudan.edu.cn (J.Z.); 21110220003@m.fudan.edu.cn (W.F.); 20110220109@fudan.edu.cn (Q.Y.); lily_zhang@fudan.edu.cn (L.Z.); yuanhangren@fudan.edu.cn (Y.R.)

**Keywords:** nanoconfined CuZn catalyst, atomic level regulation, MAS NMR, CO_2_ utilization, methanol

## Abstract

CuZn-based catalysts are widely used in CO_2_ hydrogenation, which may effectively convert CO_2_ to methanol and alleviate CO_2_ emission issues. The precise design of a model catalyst with a clear atomic structure is crucial in studying the relationship between structure and catalytic activity. In this work, a one-pot strategy was used to synthesize CuZn@ZSM-5 catalysts with approximately two Cu atoms and one Zn atom per unit cell. Atomic Cu and Zn species are confirmed to be located in the [5^4^.6.10^2^] and [6^2^.10^4^] tilings, respectively, by using magic-angle spinning nuclear magnetic resonance spectroscopy (MAS NMR), synchrotron X-ray powder diffraction (SXRD) and high-signal-to-noise-ratio annular dark field scanning transmission electron microscopy (High SNR ADF-STEM). Catalytic hydrogenation of CO_2_ to methanol was used as a model reaction to investigate the activity of the catalyst with confined active species. Compared to the Cu@ZSM-5, Zn@ZSM-5 and their mixture, the CuZn@ZSM-5 catalyst with a close Cu–Zn distance of 4.5 Å achieves a comparable methanol space–time yield (STY) of 92.0 mg_methanol_·g_catal_^−1^·h^−1^ at 533 K and 4 MPa with high stability. This method is able to confine one to three metal atoms in the zeolite channel and avoid migration and agglomeration of the atoms during the reaction, which maintains the stability of the catalyst and provides an efficient way for adjustment of the type and number of metal atoms along with the distances between them in zeolites.

## 1. Introduction

The environmental problems caused by greenhouse gas CO_2_ emissions are becoming increasingly imminent [1]. Capturing and separating CO_2_ from concentrated emission sources and using it as a C1 chemical raw material to produce methanol, formic acid, dimethyl ether and other chemicals has aroused widespread research interest [2,3,4,5]. Methanol can be used as a clean fuel or as a key chemical used to synthesize high-value-added derivatives with broad industrial prospects. Currently, the industrial process for preparing methanol is normally based on syngas as the raw material, which has been developed since 1961 [6]. With the increased importance of CO_2_ emission reduction and the shortage of petroleum resources, the CO and CO_2_ hydrogenation to methanol reaction is receiving more attention [7,8].

The synthesis of methanol from syngas or CO_2_ hydrogenation commonly uses Cu–ZnO–Al_2_O_3_ as a catalyst, where the Al_2_O_3_ is used to support the active Cu and Zn species [9]. Whether the active sites of CO_2_ hydrogenation to methanol reactions are Cu–ZnO interfacial sites or Cu–Zn alloy sites remains controversial [10,11,12]. However, the coexistence of Cu, Cu–Zn alloy and ZnO phases in catalysts prepared by impregnation or co-precipitation methods is inevitable, which causes great difficulties in the study of the relationship between the structure of catalysts and their activity [13]. In addition, due to the increase in surface energy of metal species with decreasing particle size, metal species dispersed on supports are thermodynamically unstable and prone to sintering during reaction [14]. The above factors may significantly reduce the metal dispersion and lead to rapid deactivation, limiting their practical industrial application [15]. The introduction of the confinement effect in the preparation of catalysts not only improves the regulation of catalytic active sites but also enhances phase purity and structural stability [16]. This confinement effect may be achieved by separating active metal species using zeolites [17,18,19,20,21,22], metal–organic frameworks [23,24] and two-dimensional layered materials [25] with their special porous structures, leading to the anti-sintering performance of catalysts and stability enhancement [26,27,28].

Zeolites are widely used in heterogeneous catalysis due to their unique microporous framework structures and high stability [29]. In ZSM-5, eight 5-membered ring [5^8^] tilings with *D_2d_* symmetry construct characteristic two-dimensional 10-membered ring (10MR) channels. The 10MR channels parallel to the *a*-axis direction are zigzagged with a pore size of 5.5 Å × 5.1 Å, and the 10MR channels parallel to the *b*-axis direction are straight with a pore size of 5.3 Å × 5.6 Å [30,31,32]. These channels with moderate size provide the possibility for the confinement of metals. The one-pot synthesis strategy has been used to confine metal atoms in zeolites, such as Cu-CHA [33,34], Cu@FAU [28], Pt@Silicalite-1 [20], Pt@Ge-UTL [27], etc. STEM [26], EXAFS [27] and SXRD [28] have been employed for the study of the precise position of metal atoms and metal–metal or metal–framework interactions. These efforts show that the controllable synthesis and characterization of catalysts, especially the precise positioning of metal atoms, are particularly important for studying the structure–activity relationship of catalysts.

In this work, a series of M@ZSM-5 catalysts (M represents Cu, Zn, CuZn) were prepared by a one-pot synthesis method. The metals with close distances were located in specific positions in the zeolite channels using MAS NMR, SXRD and High SNR ADF-STEM techniques. The catalytic activity of Cu and Zn in M@ZSM-5 catalysts was investigated in CO_2_ hydrogenation to methanol reactions. The synergistic effect of different mixing modes for M@ZSM-5 catalysts on the catalytic activity was also studied.

## 2. Materials and Methods

### 2.1. Materials

Sodium aluminate (Al_2_O_3_ = 52.82%, Na_2_O = 40.24%, Alfa Aesar, Waltham, MA, USA), tetraethylorthosilicate (TEOS, SiO_2_ = 28%, Cross, Houston, TX, USA), sodium hydroxide (NaOH, ≥98%, Greagent, Shanghai, China), copper nitrate trihydrate (Cu(NO_3_)_2_·3H_2_O, 99%, Sinopharm, Shanghai, China) and zinc nitrate hexahydrate (Zn(NO_3_)_2_·6H_2_O, 99%, Sinopharm) were used as Al, Si, Na, Cu and Zn sources, respectively, while tetrapropylammonium hydroxide aqueous solution (TPAOH, 40 wt.%, Energy chemical, Shanghai, China) was employed as a structure-directing agent along with ethylenediamine (EDA, 99%, Sinopharm, Shanghai, China). All the reagents were used without further purification.

### 2.2. Catalyst Synthesis

ZSM-5 samples were obtained using a hydrothermal method with a composition of 0.5 Al_2_O_3_:100 SiO_2_:20 TPAOH:4 Na_2_O:2500 H_2_O [35]. In a typical synthesis, 0.13 g of NaOH and 19.45 g of deionized water were weighed in a 100 mL beaker in sequence, 0.05 g of sodium aluminate and 5.08 g of TPAOH were added with stirring, and then 10.71 g of TEOS was added to this solution and stirred for more than 4 h. The final gel was transferred to a 100 mL Teflon-lined autoclave, heated at a rate of 2 K·min^−1^ to 443 K, kept at this temperature for 72 h, and cooled naturally to room temperature. The product was separated by centrifugation and washed with deionized water and ethanol. Finally, the powder sample was calcined in a muffle furnace at 823 K for 6 h.

The compositions for Cu@ZSM-5, Zn@ZSM-5 and CuZn@ZSM-5 were 0.5 Al_2_O_3_:100 SiO_2_:20 TPAOH:4 Na_2_O:2500 H_2_O:2 Cu(NO_3_)_2_:4 EDA, 0.5 Al_2_O_3_:100 SiO_2_:20 TPAOH:4 Na_2_O:2500 H_2_O:1 Zn(NO_3_)_2_:3 EDA and 0.5 Al_2_O_3_:100 SiO_2_:20 TPAOH:4 Na_2_O:2500 H_2_O:2 Cu(NO_3_)_2_:1 Zn(NO_3_)_2_:7 EDA, respectively. An amount of 0.24 g of Cu(NO_3_)_2_·3H_2_O or/and 0.10 g of Zn(NO_3_)_2_·6H_2_O were weighed in a 50 mL beaker, and 5 g of deionized water was added. A transparent solution was formed after a desired amount of EDA was slowly added. The solution was then added dropwise to the above gel for the synthesis of ZSM-5 and finally TEOS was added. The remaining procedures were the same as that for the synthesis of ZSM-5 [20,35].

### 2.3. Characterization Techniques

X-ray diffraction (XRD) patterns were recorded on a Bruker D2 PHASER Advance diffractometer (Brucker, Madison, WI, USA), operated at 30 kV and 10 mA using Cu Kα (λ = 1.5418 Å) with a step width of 0.01° and a step time of 0.2 s. High-resolution SXRD data were obtained from MYTHEN detectors on a BL02B2 SPring-8 Japan [36] and refined by the Rietveld method using TOPAS refinement software (TOPAS-Academic V7). The patterns were collected in the 2*θ* range of 2–80° with 0.006° steps. Each pattern was collected for 4 min. In total, there were more than 800 *hkl* reflections measured, of which at least 300 independent *hkl* reflections were observed.

High SNR ADF-STEM images were captured on a JEM-ARM300F GRAND ARM (JEOL, Tokyo, Japan) aberration-corrected scanning transmission electron microscope equipped with double aberration correctors with an operating voltage of 300 kV and camera length of 800 mm. Image acquisition according to methods reported in the literature was conducted with a semi-angle of 10 mard, collection angles of 10–40 mard and an electron dose of 2653 e^−^/Å^2^ [37]. High-resolution transmission electron microscope photographs (HR-TEM) were obtained and selected-area electron diffraction (SAED) and elemental analysis (EDX) were performed using the Tecnai G2 F20 S-Twin (FEI, Hillsboro, OR, USA) field emission transmission electron microscope operated at 200 kV and equipped with an Oxford Instruments X-Max 80T EDS (Oxford Instruments, Abingdon, Oxfordshire, UK) probe.

Solid-state MAS NMR experiments were recorded using a Bruker AVANCE III 400WB NMR spectroscope (Brucker, Madison, WI, USA) at 9.4 T equipped with BL4 and BL7 MAS DVT probes (Brucker, Madison, WI, USA) using zirconia rotors with Kel-F caps. ^27^Al MAS NMR spectra were obtained at 104.3 MHz using a 4 mm rotor with a spinning rate of 12 kHz. A single-pulse sequence was used with a 10° pulse (0.13 μs) and a recycle delay of 0.3 s. The powder zeolite samples were balanced at the desired humidity in a glass container over a saturated MgCl_2_ aqueous solution for more than 24 h prior to ^27^Al NMR measurements. ^29^Si MAS NMR spectra were obtained at 79.6 MHz using a 7 mm rotor with a spinning rate of 4 kHz. A single-pulse sequence was used with a 90° pulse (4.3 μs) and a recycle delay of 180 s. ^1^H-^29^Si CP/MAS NMR spectra were recorded with a 90° pulse (4.3 μs), a contact time of 1–18 ms and a recycle delay of 2 s. A 1 M Al(NO_3_)_3_ aqueous solution and tetramethylsilane (Si(CH_3_)_4_, TMS) were used as the ^27^Al and ^29^Si external chemical shift standards, respectively.

X-ray photoelectron spectroscopy (XPS) was performed using a PHI PHI5000C instrument equipped with a Versa probe and a Mg Kα emission light source (*h*v = 1253.6 eV) at 15 kV and 20 mA. Binding energy was calibrated with the contaminated carbon C 1s of 284.8 eV.

A N_2_ adsorption–desorption study was carried out on a Micromeritics ASAP 2020 Plus analyzer (Micromeritics, Norcross, GA, USA) at 77 K. The samples were pretreated at 573 K and 1.3 × 10^−2^ Pa for 10 h. The specific surface area was obtained using the Brunauer–Emmett–Teller (BET) equation and the micropore volume and micropore surface areas were calculated using a *t*-plot, and the pore size distribution was calculated using the Barrett–Joyner–Halenda model.

H_2_ temperature-programmed reduction (H_2_-TPR) was conducted using a Micromeritics ChemiSorb 2720 chemical adsorption instrument (Micromeritics, Norcross, GA, USA) with a thermal conductivity detector (TCD). A typical procedure was as follows: 100 mg of sample was loaded into a quartz U-tube and pretreated with He at 50 mL·min^−1^. The temperature was raised at a rate of 10 K·min^−1^ from ambient temperature to 473 K and maintained for 1 h and then lowered to below 317 K. The gas was switched to 10% H_2_/Ar and the temperature was raised at a rate of 10 K·min^−1^ to 923 K. Inductively coupled plasma atomic emission spectroscopy (ICP-AES) was performed using a Varian 725-ES plasma emission spectrometer.

### 2.4. Catalytic Tests

A stainless-steel reactor was equipped with a quartz tube liner with an inner diameter of 8 m and a length of 58 cm. A total of 0.1 g of 40–60 mesh catalyst was loaded with a layer height of about 0.5 cm. The reaction was carried out within the temperature range of 513–593 K, at a pressure of 4.0 MPa, a reaction gas composition H_2_/CO_2_/Ar of 72/24/4, a reaction gas flow rate of 40 mL·min^−1^ and a space velocity GHSV of 24,000 mL·g_catal_^−1^·h^−1^.

A Huaai GC-9560 gas chromatograph (Shanghai Huaai Chromatographic Analysis Technology Co., Ltd., Shanghai, China) was equipped with a TCD and a flame ion detector (FID) for the analysis of products. CO_2_, CO, Ar, CH_4_ and some other products were analyzed with the TCD through a Porapak N-filled column (5 m × 2 mm) and a 13X zeolite-filled column (3 m × 2 mm). Light olefins and methanol were analyzed with the FID through an HP-PLOT Al_2_O_3_ capillary column (50 m × 0.32 mm × 10 μm). The conversion of CO_2_, the selectivity of products, the space–time yield (STY) of methanol and the apparent turnover frequency (TOF) for methanol were calculated as follows:

(1) $${\mathrm{Conv}.\;}_{{\mathrm{CO}}_{2}}\;(\%)=\frac{n_{{\mathrm{CO}}_{2}-\mathrm{in}}\;-\;n_{{\mathrm{CO}}_{2}-\mathrm{out}}}{n_{{\mathrm{CO}}_{2}-\mathrm{in}}}\;\text{×}\;100\%$$

(2) $${\mathrm{Select}.\;}_{C_{i}H_{j}O_{k}}\;(\%)=\frac{i\;\text{×}\;n_{C_{i}H_{j}O_{k}}}{\sum i\;\text{×}\;n_{C_{i}H_{j}O_{k}}}\;\text{×}\;100\%$$

(3) $${\mathrm{STY}}_{\mathrm{methanol}}=\frac{\mathrm{GHSV}\;\text{×}\;\mathrm{Conv}.\;\text{×}\;\mathrm{Select}.\;\text{×}\;32\;\text{×}\;0.24}{22.4}\;({\mathrm{mg}}_{\mathrm{methanol}}\text{·}g_{\mathrm{catal}}^{-1}\text{·}h^{-1})$$

(4) $${\mathrm{TOF}}_{\mathrm{methanol}}=\frac{\mathrm{GHSV}\;\text{×}\;\mathrm{Conv}.\;\text{×}\;\mathrm{Select}.\;\text{×}\;0.24}{22.4\;\text{×}\;1000\;\text{×}\;(\frac{\mathrm{Cu}\;\mathrm{wt}.\%}{64}+\frac{\mathrm{Zn}\;\mathrm{wt}.\%}{65})}\;(h^{-1})$$

## 3. Results

### 3.1. Structural Characterization

**Figure 1:**
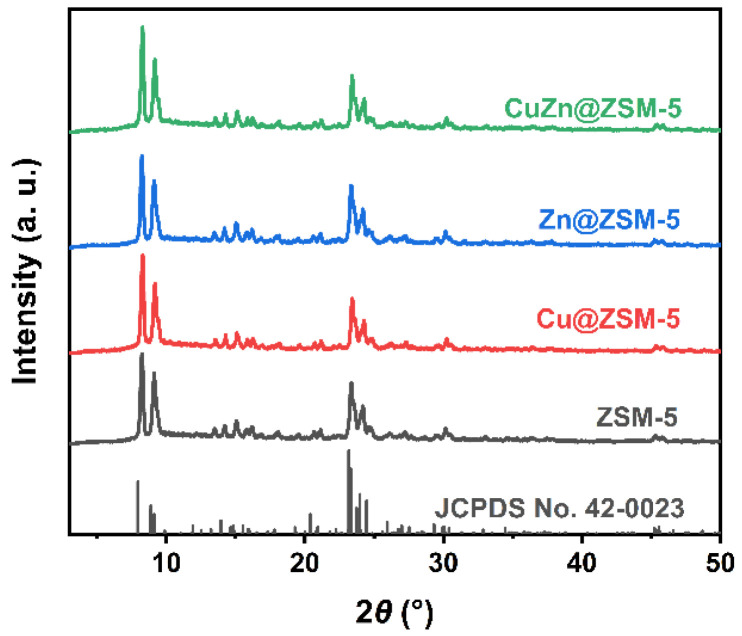
XRD patterns of ZSM-5, Cu@ZSM-5, Zn@ZSM-5 and CuZn@ZSM-5.

The XRD patterns show that all ZSM-5, Cu@ZSM-5, Zn@ZSM-5 and CuZn@ZSM-5 synthesized by the hydrothermal method have typical MFI structure (JCPDS No. 42-0023) without any impurity (Figure 1). No diffraction peaks assigned to Cu or Zn or their oxides are observed.

**Figure 2:**
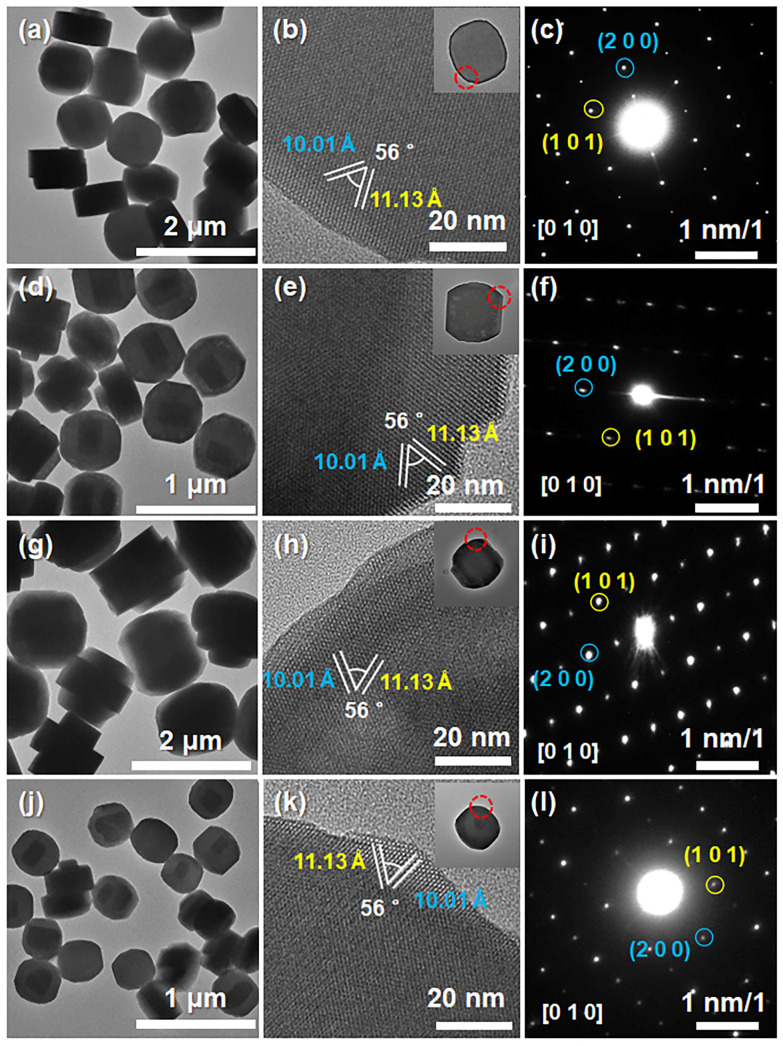
TEM, HRTEM, and SAED images of (**a**–**c**) ZSM-5, (**d**–**f**) Cu@ZSM-5, (**g**–**i**) Zn@ZSM-5 and (**j**–**l**) CuZn@ZSM-5 (The red circle represents the HRTEM position).

The morphology and structure of the synthesized zeolites were studied by TEM. Figure 2 shows the TEM, HRTEM and SAED images of the synthesized ZSM-5, Cu@ZSM-5, Zn@ZSM-5 and CuZn@ZSM-5. All samples exhibit a hexagonal prism with a short *b* axis. The average particle size and thickness along the *b* axis of ZSM-5, Cu@ZSM-5, Zn@ZSM-5, and CuZn@ZSM-5 are about 1 μm and 200 nm, 650 nm and 150 nm, 1.2 μm and 400 nm, and 200 nm and 80 nm, respectively. All samples are uniform in shape and size. From the HETEM images (Figure 2b,e,h,k), lattice fringes along the direction of [010] may be observed in all samples. Combined with the corresponding SAED images (Figure 2c,f,i,l), the lattice spacings of 10.01 and 11.13 Å correspond to the interplanar spacings of (200) and (101), respectively, and the angle between (200) and (101) planes is 56° [38].

The EDS mapping results (Figure S1) show that all elements in the samples are evenly distributed and no large-scale aggregation of Cu or Zn is observed. This indicates that Cu and Zn are well dispersed in zeolite framework or channels. The composition of the samples was determined by ICP-AES. The Si/Al ratio of ZSM-5, Cu@ZSM-5, Zn@ZSM-5 and CuZn@ZSM-5 are 86, 79, 82 and 75, respectively, which are close to the Si/Al of 100 in the starting synthesis gel. The contents of metals in the samples are shown in Table 1 and are close to those in the starting synthesis gel.

Table 2 shows texture data of the products after calcination at 823 K for the removal of template molecules in the channels as part of the N_2_ adsorption–desorption experiments. All samples have typical microporous structures with the largest microporous specific surface area of 337 m^2^·g^−1^ found in ZSM-5. After introducing the metal species, the microporous specific surface areas decrease significantly and the smallest microporous specific surface area of CuZn@ZSM-5 is 193 m^2^·g^−1^. ZSM-5 has the maximum microporous volume of 0.13 cm^3^·g^−1^ among the four samples when compared with Cu@ZSM-5, Zn@ZSM-5 and CuZn@ZSM-5 which decrease by 0.03, 0.01 and 0.06 cm^3^·g^−1^, respectively. The MFI structural units form 10MR with a pore size of about 5.1–5.6 Å and 5MR with a size of about 2.6–3.1 Å. Since the kinetic diameter of N_2_ molecules is 3.04 Å, N_2_ adsorption can only detect the 10MR channels within the framework [39]. Through the trend change of microporous specific surface area and volume, it can be speculated that the confined metal Cu or/and Zn are located in the 10MR channel of the MFI framework. The confined metal species located in the microporous channels of the zeolite occupy part of the microporous space, affecting the adsorption of N_2_ molecules in the microporous channels and leading to a decrease in microporous volume and specific surface area.

**Figure 3:**
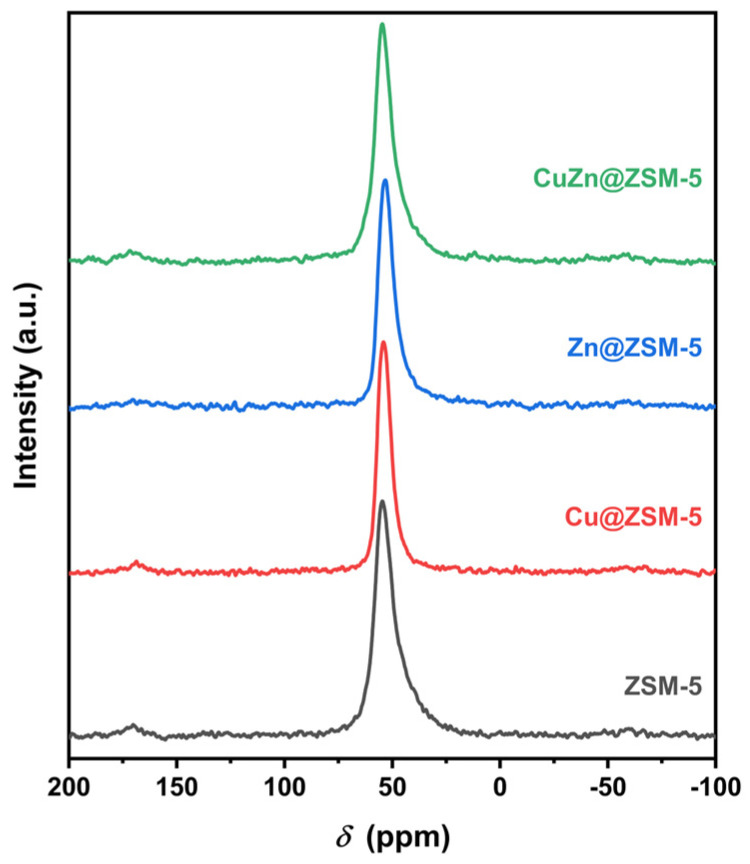
^27^Al MAS NMR spectra of ZSM-5, Cu@ZSM-5, Zn@ZSM-5 and CuZn@ZSM-5.

The zeolite framework structure was further studied by MAS NMR. Figure 3 shows that the ^27^Al MAS NMR spectra of all samples have a sharp peak at 55 ppm, attributed to the four-coordinate framework aluminum. No peak is found at 0 ppm, indicating that the samples do not contain six-coordinate extra-framework aluminum [40].

**Figure 4:**
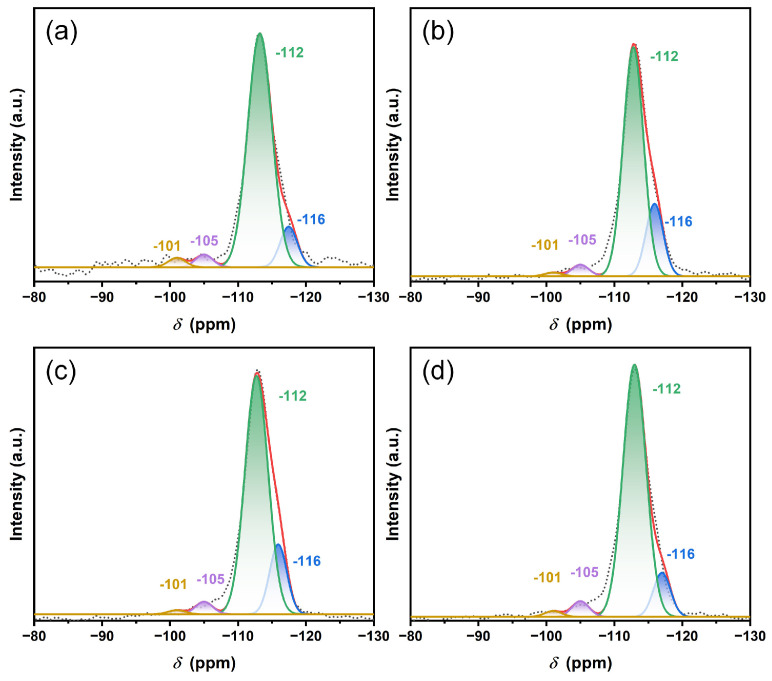
Single-pulse ^29^Si MAS NMR spectra of (**a**) ZSM-5, (**b**) Cu@ZSM-5, (**c**) Zn@ZSM-5 and (**d**) CuZn@ZSM-5 after the removal of the template by calcination. (The dashed line represents the experimental results and the red line represents the fitted accumulated results.)

**Figure 5:**
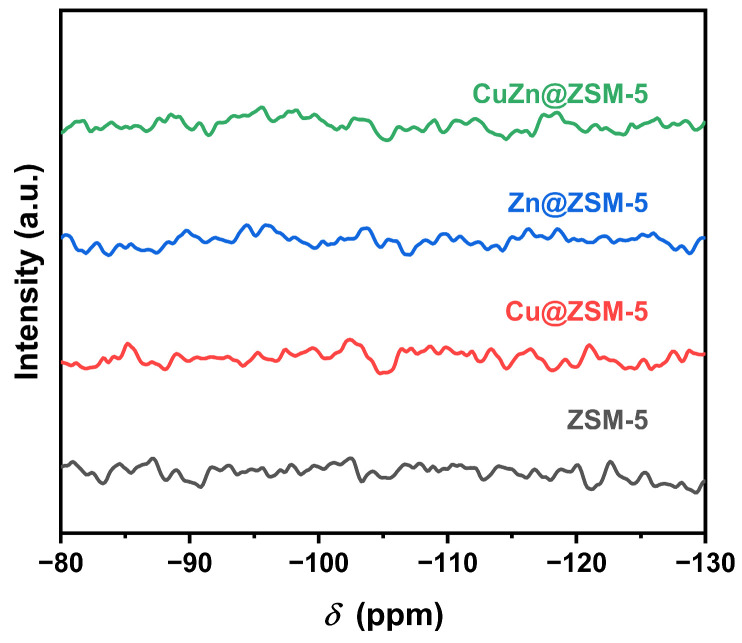
^1^H-^29^Si CP/MAS NMR spectra of ZSM-5, Cu@ZSM-5, Zn@ZSM-5 and CuZn@ZSM-5 with a contact time of 8 ms after the removal of the template by calcination.

Figure 4 and Figure 5 show the ^29^Si MAS NMR and ^1^H-^29^Si CP/MAS NMR spectra of ZSM-5, Cu@ZSM-5, Zn@ZSM-5 and CuZn@ZSM-5. In the ^29^Si MAS NMR spectra, all zeolites show four peaks located at −101, −105, −112 and −116 ppm. The peak at −101 ppm can be attributed to Q^3^ (Si(OSi)_3_(OAl)_1_ or Si(OSi)_3_OH), the peak at −105 ppm can be attributed to Q^3^ (Si(OSi)_3_(OAl)_1_), and the peaks at −112 and −116 ppm can be attributed to Q^4^ (Si(OSi)_4_) [41]. However, in the ^1^H-^29^Si CP/MAS NMR spectra, no NMR peak appears in the chemical shift range of −80~−120 ppm, indicating that the MFI framework has no hydroxyl defects and is intact after calcination. Therefore, the peak at −101 ppm in Figure 4 can be attributed to Si(OSi)_3_(OAl)_1_ rather than Si(OSi)_3_OH [42]. Since there are no amorphous Si species and strong XRD diffraction peaks, the synthesized products have high crystallinity. By fitting the peaks of each spectrum in Figure 4, Si/Al may be calculated and is listed in Table 3, which is basically consistent with the ICP-AES results.

### 3.2. Insight into the Confined Position of the Metal in ZSM-5

Since NMR results reflect the local environment of the Si atoms, different local environments of Si, particularly affected by metal introduced in the zeolite framework, may lead to a change in full-width-at-half-maximum (FWHM) even with the same Si/Al ratio. The deconvolution results of the ^29^Si MAS NMR spectra for ZSM-5, Cu@ZSM-5, Zn@ZSM-5 and CuZn@ZSM-5 samples indicate similar FWHM for their corresponding peaks. Therefore, no heteroatom is introduced into the MFI framework [43]. In addition, if heteroatoms Cu or/and Zn are introduced into the MFI framework, the MFI framework forms defects, leading to the formation Si(OSi)_3_OH. This structural change may cause cross-polarization from H to Si, thus enhancing ^1^H-^29^Si CP/MAS NMR intensities. As seen in Figure 5, there is no ^1^H-^29^Si CP/MAS NMR peak in the range of −80~−120 ppm, indicating that there is no Si(OSi)_3_OH in the MFI framework after calcination and no heteroatoms have been introduced into the MFI framework.

**Figure 6:**
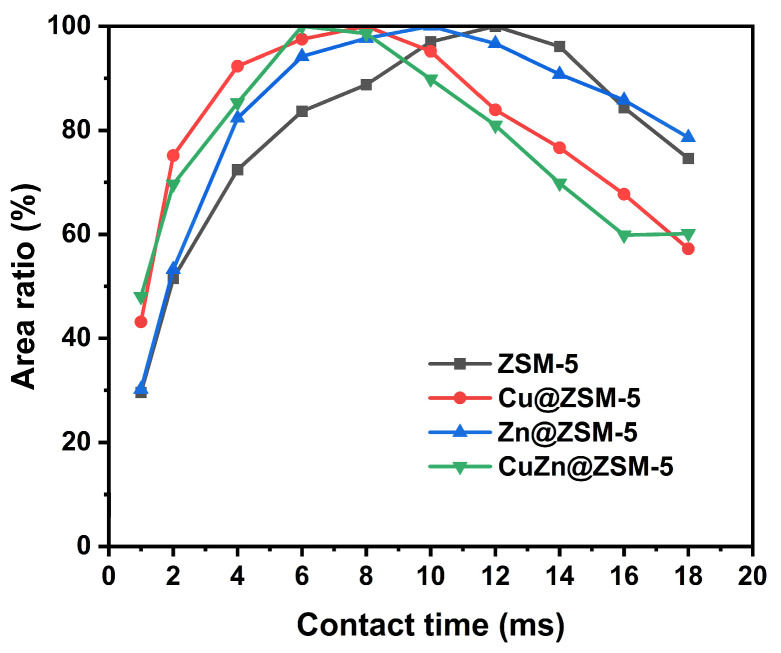
Area ratio of the peak at −112 ppm to the total peak area from variable contact-time ^1^H-^29^Si CP/MAS NMR spectra for ZSM-5, Cu@ZSM-5, Zn@ZSM-5 and CuZn@ZSM-5.

To further study the structural relationship between the MFI framework and the introduced Cu or/and Zn species, a series of variable contact-time ^1^H-^29^Si CP/MAS NMR experiments were employed using the as-prepared (uncalcined) ZSM-5, Cu@ZSM-5, Zn@ZSM-5 and CuZn@ZSM-5 samples. As shown in Figures S2–S5, the intensities of all peaks at −101, −105, −112 and −116 ppm reach a maximum at certain contact times, among which the peak at −112 ppm is the strongest and its intensity change with contact time is displayed in Figure 6. The contact times at the highest peak intensity for ZSM-5, Cu@ZSM-5, Zn@ZSM-5 and CuZn@ZSM-5 are 12, 8, 10 and 6 ms, respectively. Although there is no heteroatom introduced into the MFI framework to form Si(OSi)_3_OH, the structure-directing agent containing H atoms in the zeolite channels may enhance the ^1^H-^29^Si cross-polarization. In addition, the existence of confined Cu or/and Zn in the channels may further reduce the motion of H atoms and the distance between H and Si, thus reducing the optimal CP contact time [44,45,46], which is consistent with the results shown in Figure 6. Therefore, the various contact-time ^1^H-^29^Si CP/MAS NMR experiments clearly show the confinement of Cu or/and Zn in the ZSM-5 channels.

**Figure 7:**
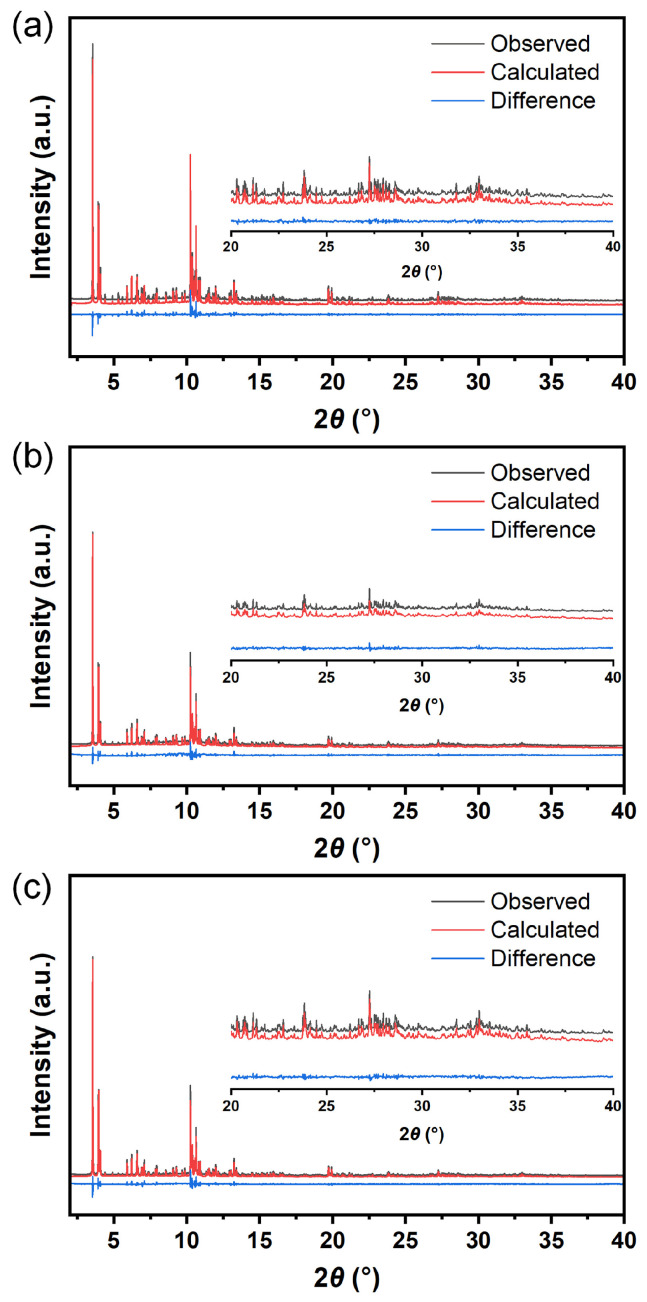
SXRD experimental patterns (black lines), fitted patterns (red lines) and their difference (blue lines) for (**a**) Cu@ZSM-5, (**b**) Zn@ZSM-5 and (**c**) CuZn@ZSM-5.

In order to have a better understanding of the location of the confined metals in the zeolite structure at the atomic level, synchrotron X-ray diffraction was carried out to obtain SXRD patterns (Figure 7) for the samples of Cu@ZSM-5, Zn@ZSM-5 and CuZn@ZSM-5 [36]. The refined structures of the samples were obtained by the Rietveld method using TOPAS software (TOPAS-Academic V7), and the fitted crystallographic parameters are shown in Table S1, and atomic information from the Rietveld refinement of the samples are listed in Tables S2–S4. According to the fitting results, it can be seen that the *Rwp* of Cu@ZSM-5, Zn@ZSM-5 and CuZn@ZSM-5 are 7.59%, 9.23% and 9.69%, respectively, indicating the high credibility of the fitting results [47]. The inset patterns in Figure 7 show that the error line fluctuates slightly in the 2*θ* range of 20–40°, reflecting the high quality and accuracy of refining local structures [39]. Combined with the ICP-AES results, the average contents of Cu, Zn and CuZn per unit cell of Cu@ZSM-5, Zn@ZSM-5 and CuZn@ZSM-5 are 1.82, 0.88 and 2.34/0.89, respectively.

**Figure 8:**
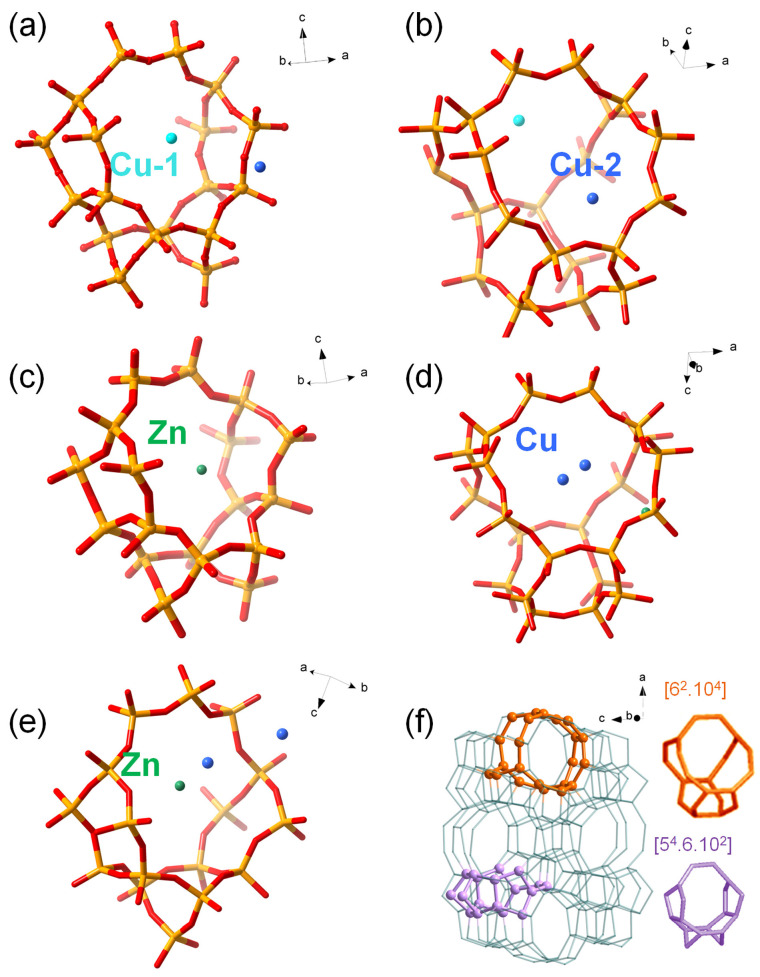
The relationship between Cu, Zn and MFI framework calculated by SXRD. (**a**) Cu-1 in [5^4^.6.10^2^] tiling of Cu@ZSM-5, (**b**) Cu-2 in [6^2^.10^4^] tiling of Cu@ZSM-5, (**c**) Zn in [5^4^.6.10^2^] tiling of Zn@ZSM-5, (**d**) Cu in [6^2^.10^4^] tiling of CuZn@ZSM-5, (**e**) Zn in [5^4^.6.10^2^] tiling of CuZn@ZSM-5. (**f**) Positions of [5^4^.6.10^2^] (purple) and [6^2^.10^4^] (orange) tilings in the MFI framework.

Based on the refined structures (Tables S2–S4), the positional relationship between the confined metal atoms and the MFI framework in Cu@ZSM-5, Zn@ZSM-5 and CuZn@ZSM-5 are shown in Figure 8. According to the probability distribution of confined metal from the structural model obtained by SXRD analysis, there are two different positions of Cu in the Cu@ZSM-5 sample. Cu-1 is located in the [5^4^.6.10^2^] tiling (Figure 8a), and the shortest Cu–O distance between Cu-1 and the surrounding framework structure is 3.9 Å. Cu-2 is located in the [6^2^.10^4^] tiling (Figure 8b), and the shortest Cu–O distance between Cu-2 and the surrounding framework structure is 2.7 Å. The ratio of Cu-1 to Cu-2 is 1.20. Zn in the Zn@ZSM-5 is located in the [5^4^.6.10^2^] tiling (Figure 8c), and the shortest Zn–O distance to the surrounding framework structure is 3.3 Å. There are two types of Cu atoms and one type of Zn atom in the CuZn@ZSM-5 sample. Both Cu atoms are located in the unit composed of the [6^2^.10^4^] tiling (Figure 8d). The distance between two Cu atoms is 5.2 Å. Zn is located in the unit composed of the [5^4^.6.10^2^] tiling (Figure 8e). The distance between Cu and Zn is 4.5–5.5 Å. The positions of the [5^4^.6.10^2^] tiling and the [6^2^.10^4^] tiling in the MFI framework are shown in Figure 8f, where the [5^4^.6.10^2^] tiling is part of the sinusoidal channel, and the [6^2^.10^4^] tiling is located at the intersection of the sinusoidal channel and the straight channel. Based on the above SXRD results, combined with the N_2_ adsorption and ^1^H-^29^Si CP/MAS NMR results, it can be concluded that Cu and Zn species are confined in zeolite channels without entering the framework as heteroatoms.

**Figure 9:**
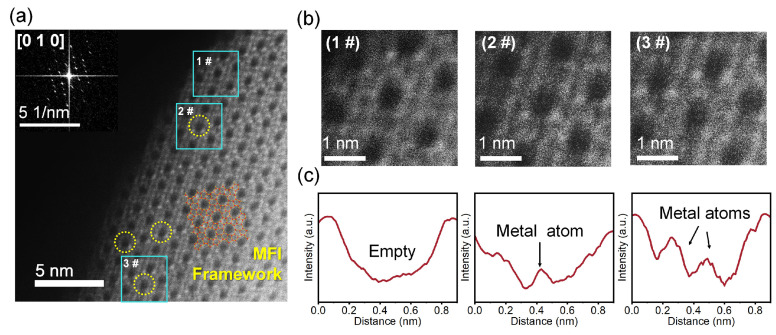
High SNR ADF-STEM image of CuZn@ZSM-5. (**a**) Representative STEM images of CuZn@ZSM-5 along the [010] zone axis, (**b**) partial enlargement of the three blue marked positions in (**a**), (**c**) intensity analysis of the pore center in (**b**).

Figure 9 shows the High SNR ADF-STEM results for CuZn@ZSM-5. The atomic -resolution image and intensity analysis provides actual spatial evidence of the zeolite framework with metal atoms in the channels [26,37]. The metal atoms in the channels of the MFI framework are directly visualized along the [010] zone axis as Figure 9a. Among them, position 1# is an empty channel, and the diameter of the hole is 5–6 Å which is consistent with the size of the 10MR of the MFI framework. The metal atoms in positions 2# and 3# can be identified by Z-contrast with an atomic radius of less than 2 Å and the distance between metal atoms and the framework is about 3–4 Å as shown in Figure 9b,c. This implies that the metal atoms occupy specific positions in the MFI channels, which is consistent with the confinement of metals mentioned above in SXRD and MAS NMR.

**Figure 10:**
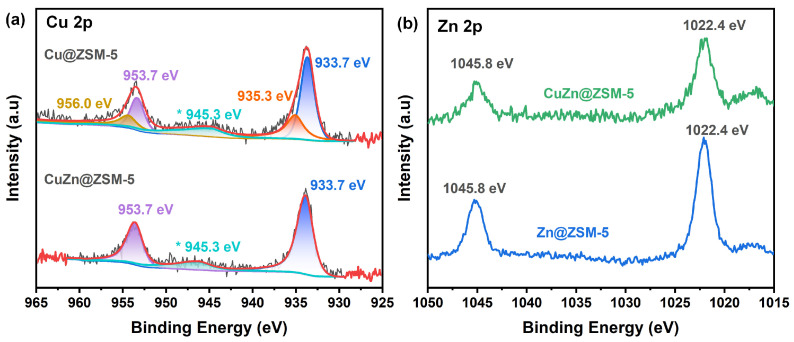
(**a**) Cu 2p and (**b**) Zn 2p XPS spectra of Cu@ZSM-5, Zn@ZSM-5 and CuZn@ZSM-5. (* Represents satellite peak.)

The valence states of the confined metals Cu and Zn were studied using XPS. Figure 10 shows the XPS spectra of Cu 2p and Zn 2p for Cu@ZSM-5, Zn@ZSM-5 and CuZn@ZSM-5 and their relevant results are shown in Table 4. The appearance of the satellite peak at 945.3 eV in the Cu 2p XPS of Cu@ZSM-5 and CuZn@ZSM-5 indicates the presence of Cu^2+^ ions. The Cu 2p_3/2_ has two peaks at 933.7 and 935.3 eV and Cu 2p_1/2_ at 953.7 and 956.0 eV, which are attributed to two different Cu^2+^ ions located in different tilings (Table S5) [48]. The area ratio of the XPS peak for Cu-2:Cu-1 in Cu@ZSM-5 is 1.25, which is close to that of the SXRD result for Cu-2:Cu-1 of 1.20. In the CuZn@ZSM-5 sample, the XPS result shows only one type of Cu. Combined with the SXRD results, two Cu^2+^ ions are located in the [6^2^.10^4^] tiling and the chemical environments of them are same. The binding energies of Zn 2p_3/2_ and 2p_1/2_ are 1022.4 and 1045.8 eV, respectively, attributed to Zn^2+^ (Table S5) [49].

H_2_-TPR (Figure S6) shows that a reduction peak appears near 505 K for Cu@ZSM-5 and CuZn@ZSM-5, which can be attributed to the reduction peak of Cu^2+^ [9]. No obvious peak related to the reduction of Zn^2+^ may be observed in Zn@ZSM-5 and CuZn@ZSM-5, indicating that Zn^2+^ is not able to be reduced below 900 K. Since there is no change in the Cu^2+^ and Zn^2+^ reduction temperatures, it indicates that there is no interaction between Cu^2+^ and Zn^2+^, which further proves that Cu^2+^ and Zn^2+^ are located in different MFI channels.

### 3.3. Catalytic Performance

**Figure 11:**
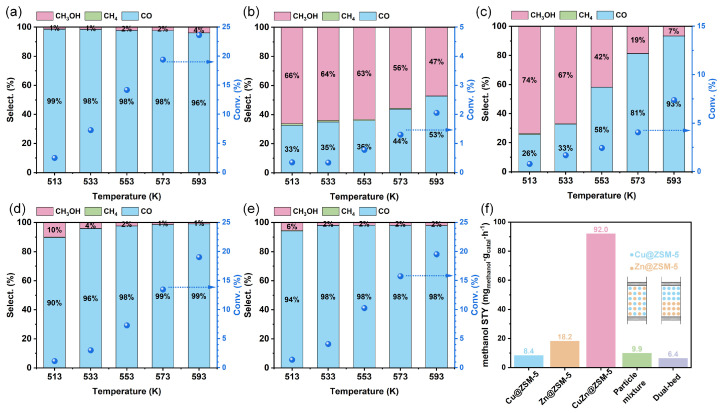
Performance of CO_2_ catalytic hydrogenation to methanol over (**a**) Cu@ZSM-5, (**b**) Zn@ZSM-5, (**c**) CuZn@ZSM-5, (**d**) mixed-particle Cu@ZSM-5 and Zn@ZSM-5 catalysts and (**e**) dual bed of Cu@ZSM-5 followed by Zn@ZSM-5 catalysts as a function of temperature, and (**f**) comparison of methanol STY over each catalyst at 533 K. Reaction conditions: 4.0 MPa, H_2_/CO_2_/Ar of 72/24/4 and GHSV of 24,000 mL·g_catal_^−1^·h^−1^.

CO_2_ hydrogenation to methanol was investigated as a model reaction under a pressure of 4.0 MPa, a H_2_/CO_2_/Ar composition of 72/24/4 and a GHSV of 24,000 mL·g_catal_^−1^·h^−1^ with a temperature range of 513–593 K. Figure 11a,b shows the results of CO_2_ hydrogenation to methanol with Cu@ZSM-5 and Zn@ZSM-5 at different temperatures. With an increase in reaction temperature from 513 K to 593 K, the CO_2_ conversion over the Cu@ZSM-5 catalyst increases from 2.48% to 23.6%, with a high CO selectivity of >96%. As reported by Kattel et al., Cu species can dissociate H_2_ rapidly, weaken CO* binding and hinder CO* hydrogenation within the temperature range of 525–575 K [11]; therefore, CO becomes the main product [50]. From 513 to 593 K, the CO_2_ conversion over the Zn@ZSM-5 catalyst is less than 3% with the methanol selectivity decreasing from 66% to 47%. Compared with Cu species, the higher oxophilicity of Zn in Zn@ZSM-5 promotes the hydrogenation of oxygenated intermediates to methanol [51,52]. With an increase in reaction temperature, the reverse water gas shift reaction intensifies, leading to a gradual increase in CO selectivity [53].

The influence of CuZn bimetallic coupling on CO_2_ hydrogenation was studied with three mixing methods: (1) CuZn@ZSM-5 catalyst with bimetallic confinement (Figure 11c), (2) Cu@ZSM-5 particles mixed with Zn@ZSM-5 particles (Figure 11d) and (3) dual bed of Cu@ZSM-5 followed by Zn@ZSM-5 (Figure 11e). The distances between Cu and Zn in the above methods (1)–(3) are <1 nm, 200–400 μm and 0.5–1 cm, respectively. As the reaction temperature increases, over the catalysts in all three systems, the conversions of CO_2_ increase and the selectivity to methanol decreases, and the selectivity to byproduct CO increases. At 533 K, the methanol STY over CuZn@ZSM-5 reaches 92.0 mg_methanol_·g_catal_·h^−1^ with a selectivity to methanol of 66.6% and a TOF for methanol of 5.90 h^−1^, while over the catalysts as described in methods (2) and (3), the methanol STY values are 9.92 and 6.38 mg_methanol_·g_catal_^−1^·h^−1^ (Figure 11f), the selectivity to methanol values are 4.04% and 1.91%, and the TOF values of methanol are 1.46 and 0.935 h^−1^, respectively. The main product for physically mixed particles with a Cu–Zn distance of 200–400 μm or dual beds with a distance of 0.5–1 cm of Cu@ZSM-5 and Zn@ZSM-5 is CO, which is similar to that of Cu@ZSM-5. Only when Cu and Zn species are in a close proximity in CuZn@ZSM-5 (<1 nm) does methanol becomes dominant in the main products along with the increase in CO_2_ conversion due to the synergistic effect of Cu and Zn species [54]. Therefore, it is expected that the close distance between Cu and Zn in CuZn@ZSM-5 may play a key role in high selective methanol formation. As the design of catalysts with confinement effects at atomic and sub-nanometer scales is still not mature, it can be further optimized by adjusting the type, distance and number of confined metals.

In addition to CO and methane, water is also a byproduct of the CO_2_ hydrogenation to methanol reaction. Under high-temperature and high-pressure conditions, the presence of water is detrimental to the stability of the zeolite framework, resulting in the removal of framework aluminum and the formation of framework defects affecting the stability of the catalyst. Figure S9 shows the XRD patterns of the CuZn@ZSM-5 catalyst before and after use for 50 h, indicating that the MFI framework remains stable. In addition, the ^27^Al MAS NMR spectra in Figure S10 further prove that there is no aluminum removed from the MFI framework during the reaction. In Figure S11a, lattice stripes with spacings of 10.01 Å and 11.13 Å and an angle of 56° between them are observed, spacings of 10.01 Å and 11.13 Å represent reflecting the (200) and (101) planes and the incident direction of [010], respectively. HAADF-STEM imagery (Figure S11b) shows that no obvious large metal clusters exist in the channels and the MFI framework structure is intact. EDS (Figure S11c) can only find well-dispersed Cu, Zn and Al elements within the MFI framework, and no agglomerate is observed. The Cu and Zn confined within the channels exhibit high stability.

## 4. Conclusions

A series of M@ZSM-5 catalysts (M represents Cu, Zn, CuZn) were prepared by a hydrothermal method. XRD, TEM and MAS NMR characterization show that the catalysts have high crystallinity without agglomerated metal particles, framework defects and extra-framework aluminum. The locations of confined metals were successfully determined by multiple characterization techniques. ^1^H-^29^Si CP/MAS NMR with variable contact time exhibit that the introduced metal species are confined in the zeolite channels with no framework of Cu and Zn species. The probability distribution structure models obtained by SXRD analysis prove that metal atoms are located at the intersection of straight and sinusoidal channels or in the sinusoidal channel of the MFI structure in M@ZSM-5 catalysts. Metal atoms in pore channels were directly observed in High SNR ADF-STEM images. This synthetic method may pave a way to fabricating stable catalysts with a single or several metal atoms in zeolite channels. In the model CO_2_ hydrogenation to methanol reaction, the CuZn@ZSM-5 catalyst with a sub-nanometer Cu–Zn distance achieves a maximum methanol STY of 92.0 mg_methanol_·g_catal_^−1^·h^−1^ at 533 K and 4 MPa. The MFI structure of CuZn@ZSM-5 remains almost constant without metal agglomeration contributed by the confinement effect of the zeolite channels after a 50 h reaction.

## Figures and Tables

**Table 1:** Metal content: theoretical values and experimental values.

Sample	Theoretical Value ^a^		Experimental Value ^b^	
	Cu (wt.%)	Zn (wt.%)	Cu (wt.%)	Zn (wt.%)
Cu@ZSM-5	2.07	-	1.82	-
Zn@ZSM-5	-	1.06	-	0.91
CuZn@ZSM-5	2.05	1.04	2.23	0.89

^a^ Calculated by starting synthesis gel. ^b^ Calculated by ICP-AES results.

**Table 2:** Texture results of the samples from N_2_ adsorption experiments.

Sample	*S*_BET_ ^a^ (m^2^·g^−1^)	*S*_micro_ (m^2^·g^−1^)	*V*_micro_ ^b^ (cm^3^·g^−1^)	*V*_total_ (cm^3^·g^−1^)	Pore Diameter ^c^ (nm)
ZSM-5	437	337	0.13	0.22	0.52
Cu@ZSM-5	410	259	0.10	0.21	0.52
Zn@ZSM-5	408	296	0.12	0.20	0.53
CuZn@ZSM-5	285	193	0.07	0.23	0.53

^a^ Calculated by the Brunauer–Emmett–Teller (BET) equation. ^b^ Calculated by t-plot method. ^c^ Calculated from NLDFT adsorption data.

**Table 3:** The different chemical environments of Si from the ^29^Si MAS NMR spectra.

Sample	Chemical Environment (%)				Si/Al * (NMR)	Si/Al (ICP-AES)
	Si(OSi)_3_(OAl)_1_		Si(OSi)_4_			
	−101 ppm	−105 ppm	−112 ppm	−116 ppm		
ZSM-5	2.4	3.2	83.9	10.5	71	86
Cu@ZSM-5	1.6	3.3	75.2	19.9	82	79
Zn@ZSM-5	1.5	3.1	78.1	17.3	87	82
CuZn@ZSM-5	1.5	3.3	83.6	11.6	83	75

* $\mathrm{Si}/\mathrm{Al}\;=\sum_{n=0}^{4}I_{\mathrm{Si}\left(\mathrm{nAl}\right)}/\sum_{n=0}^{4}0.25n[I_{\mathrm{Si}(\mathrm{nAl})}].$

**Table 4:** The different chemical environments of Cu obtained from the XPS spectra.

Sample	Chemical Environment (%)				Area Ratio of Cu-2:Cu-1
	Cu 2p_1/2_		Cu 2p_3/2_		
	Cu-1 (956.0 eV)	Cu-2 (953.7 eV)	Cu-1 (935.3 eV)	Cu-2 (933.7 eV)	
Cu@ZSM-5	12.5%	15.5%	32.0%	40.0%	1.25
CuZn@ZSM-5	-	34.1%	-	65.9%	-

## Data Availability

Data will be made available on request.

## Supplementary Materials

The following supporting information can be downloaded at: https://www.mdpi.com/article/10.3390/nano13233053/s1, Figure S1. EDS Mapping of (a) ZSM-5, (b) Cu@ZSM-5, (c) Zn@ZSM-5 and (d) CuZn@ZSM-5; Figure S2. ^1^H-^29^Si CP/MAS NMR spectra of ZSM-5 at different contact time before calcination; Figure S3. ^1^H-^29^Si CP/MAS NMR spectra of Cu@ZSM-5 at different contact time before calcination; Figure S4. ^1^H-^29^Si CP/MAS NMR spectra of Zn@ZSM-5 at different contact time before calcination; Figure S5. ^1^H-^29^Si CP/MAS NMR spectra of CuZn@ZSM-5 at different contact time before calcination; Figure S6. H_2_-TPR curves of (a) ZSM-5, (b) Cu@ZSM-5, (c) Zn@ZSM-5 and (d) CuZn@ZSM-5; Figure S7. XRD patterns of CuZn@ZSM-5 catalyst before and after 50 h reaction; Figure S8. ^27^Al MAS NMR spectra of CuZn@ZSM-5 catalyst before and after 50 h reaction; Figure S9. (a) HRTEM and SAED (inset), (b) HAADF-STEM and (c) EDS of CuZn@ZSM-5 after 50 h reaction; Table S1: Crystallographic and TOPAS fitting parameters. Table S2. Atomic information from the Rietveld refinement of Cu@ZSM-5. Table S3. Atomic information from the Rietveld refinement of Zn@ZSM-5. Table S4. Atomic information from the Rietveld refinement of CuZn@ZSM-5. Table S5. Binding energy of Cu 2p_3/2_ and Zn 2p_3/2_ [48,49].
