# Supplementary material for: Precise Confinement and Position Distribution of Atomic Cu and Zn in ZSM-5 for CO2 Hydrogenation to Methanol
Source: Nanomaterials (Basel). 2023 Nov 29;13(23):3053. doi: 10.3390/nano13233053 (PMC10708405; doi:10.3390/nano13233053)
Supplement: Supplementary file 1 [file nanomaterials-13-03053-s001.zip › nanomaterials-2736503-supplementary.pdf]

Supplementary Materials

Precise Confinement and Position Distribution of Atomic Cu and Zn in ZSM-5 for CO_2 Hydrogenation to Methanol

Precise Confinement and Position Distribution of Atomic Cu and Zn in ZSM-5 for CO_2 Hydrogenation to Methanol

Hongxin Ding, Jinwen Zhang, Wenhua Feng, Qingying Yao, Li Zhang, Yuanhang Ren, Lin Ye *, Bin Yue * and Heyong He *

Shanghai Key Laboratory of Molecular Catalysis and Innovative Materials, Department of Chemistry, Fudan University, Shanghai 200438, China; 19110220065@fudan.edu.cn (H.D.); 19110220077@fudan.edu.cn (J.Z.); 21110220003@m.fudan.edu.cn (W.F.); 20110220109@fudan.edu.cn (Q.Y.); lily_zhang@fudan.edu.cn (L.Z.); yuanhangren@fudan.edu.cn (Y.R.)

* Correspondence: yelin@fudan.edu.cn (L.Y.); yuebin@fudan.edu.cn (B.Y.); heyonghe@fudan.edu.cn (H.H.); Tel.: +86-21-3124-3916 (H.H.); Fax: +86-21-3124-5572 (H.H.)

Tabel of content

Figure S1 EDS Mapping of (a) ZSM-5, (b) Cu@ZSM-5, (c) Zn@ZSM-5 and (d) CuZn@ZSM-5. ········ S3

Figure S2 ^{1}H-^{29}Si CP/MAS NMR spectra of ZSM-5 at different contact time before calcination. ········ S4

Figure S3 ^{1}H-^{29}Si CP/MAS NMR spectra of Cu@ZSM-5 at different contact time before calcination. ········ S5

Figure S4 ^{1}H-^{29}Si CP/MAS NMR spectra of Zn@ZSM-5 at different contact time before calcination. ········ S6

Figure S5 ^{1}H-^{29}Si CP/MAS NMR spectra of CuZn@ZSM-5 at different contact time before calcination. ········ S7

Figure S6 H_2-TPR curves of (a) ZSM-5, (b) Cu@ZSM-5, (c) Zn@ZSM-5 and (d) CuZn@ZSM-5. ········ S8

Figure S7 XRD patterns of CuZn@ZSM-5 catalyst before and after 50 h reaction. ········ S9

Figure S8 ^{27}Al MAS NMR spectra of CuZn@ZSM-5 catalyst before and after 50 h reaction. ········ S10

Figure S9 (a) HRTEM and SAED (inset), (b) HAADF-STEM and (c) EDS of CuZn@ZSM-5 after 50 h reaction. ········ S11

Table S1 Crystallographic and TOPAS fitting parameters. ········ S12

Table S2 Atomic information from the Rietveld refinement of Cu@ZSM-5. ···· S13

Table S3 Atomic information from the Rietveld refinement of Zn@ZSM-5. ····· S15

Table S4 Atomic information from the Rietveld refinement of CuZn@ZSM-5. S17

Table S5 Binding energy of Cu $2p_{3/2}$ and Zn $2p_{3/2}$. ········ S19

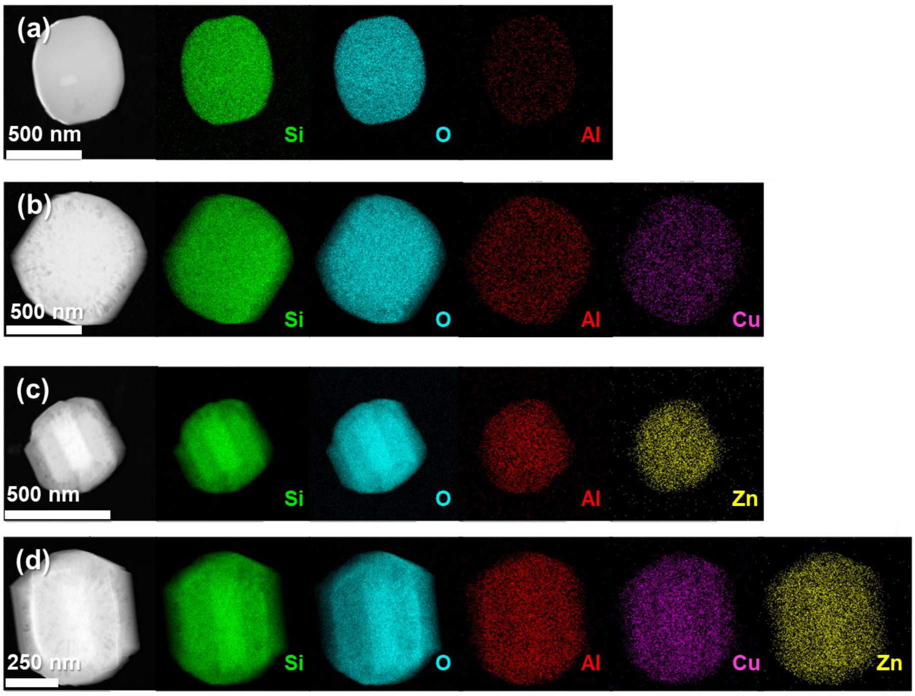

Figure S1. EDS Mapping of (a) ZSM-5, (b) Cu@ZSM-5, (c) Zn@ZSM-5 and (d) CuZn@ZSM-5.

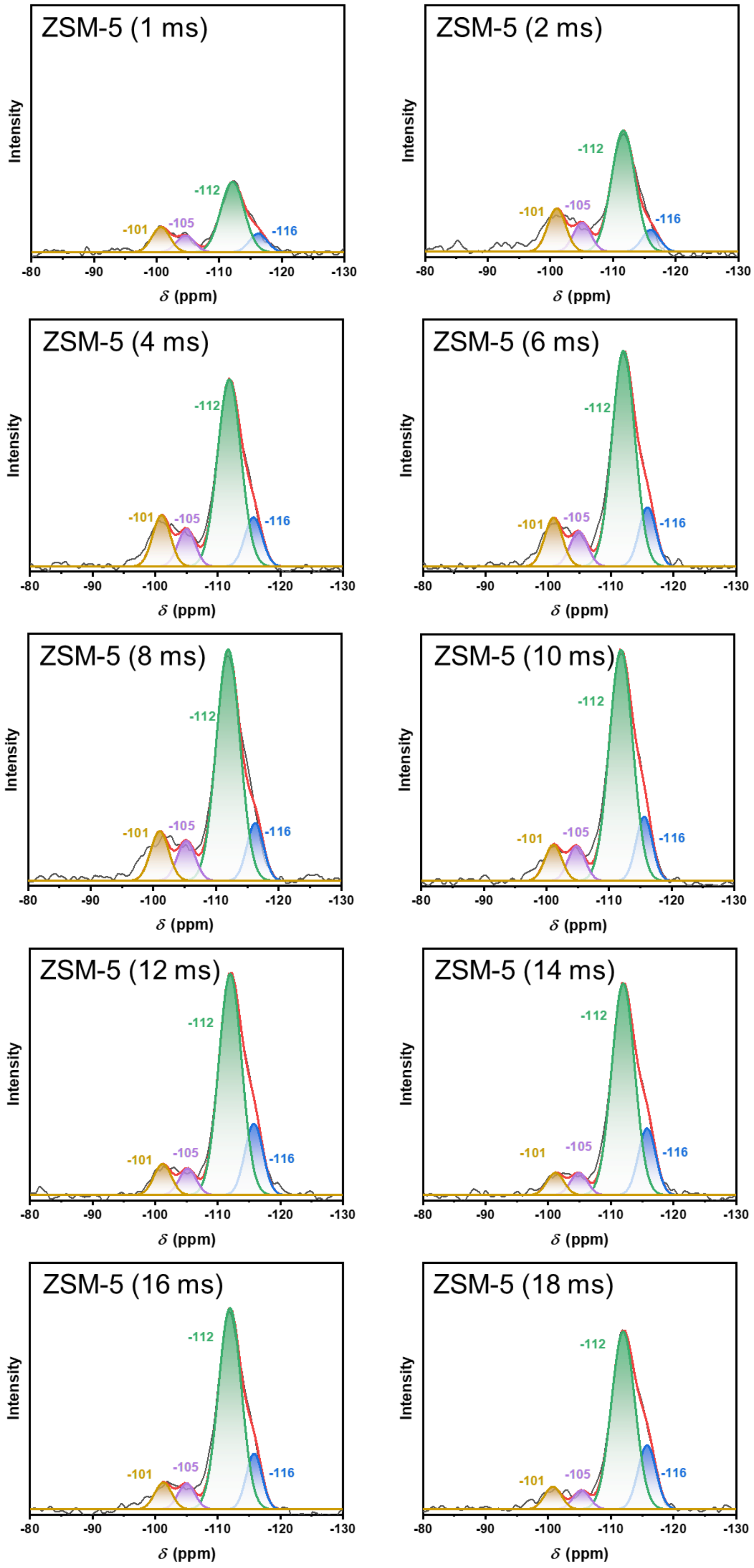

Figure S2. ^{1}H-^{29}Si CP/MAS NMR spectra of ZSM-5 at different contact time before calcination.

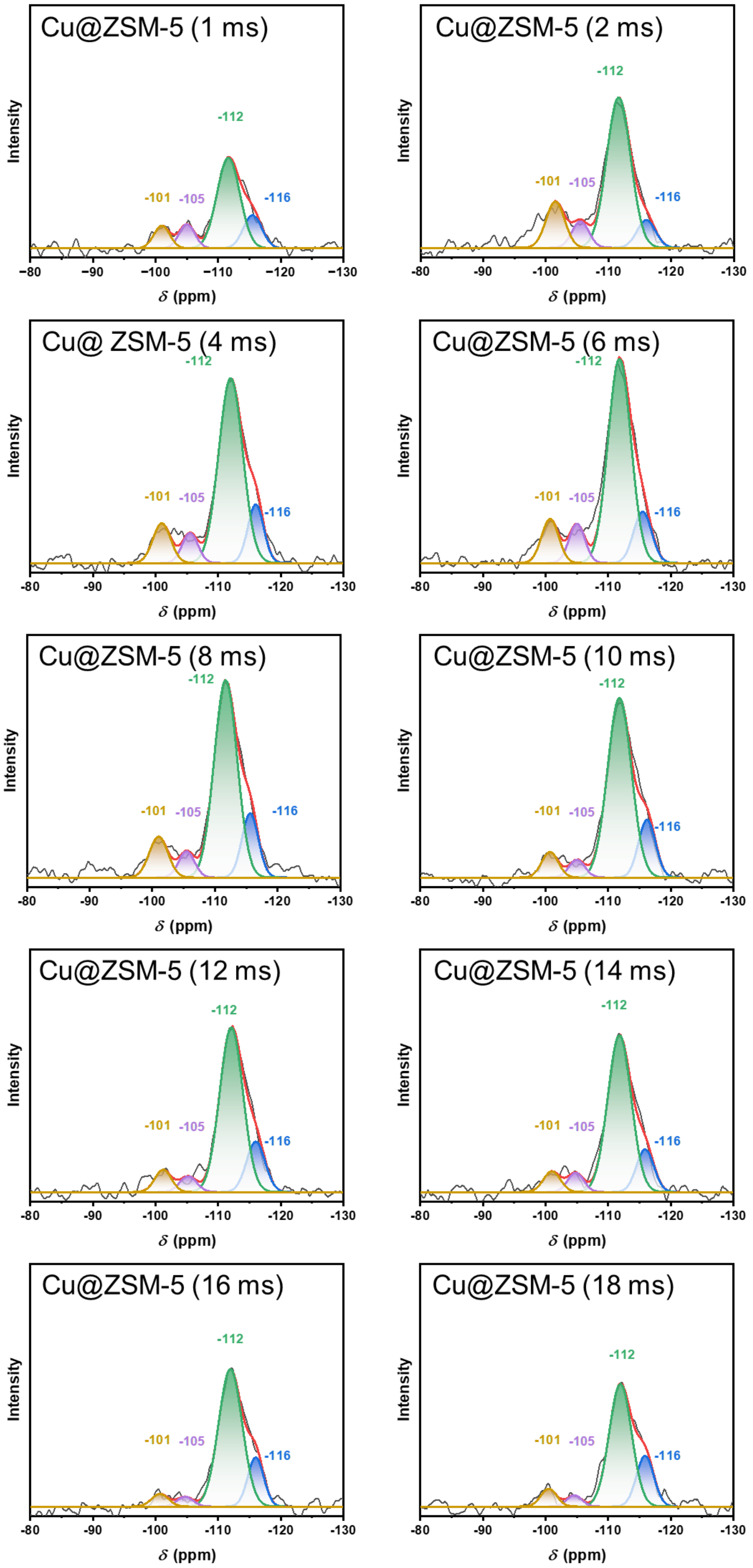

Figure S3. ^{1}H-^{29}Si CP/MAS NMR spectra of Cu@ZSM-5 at different contact time before calcination.

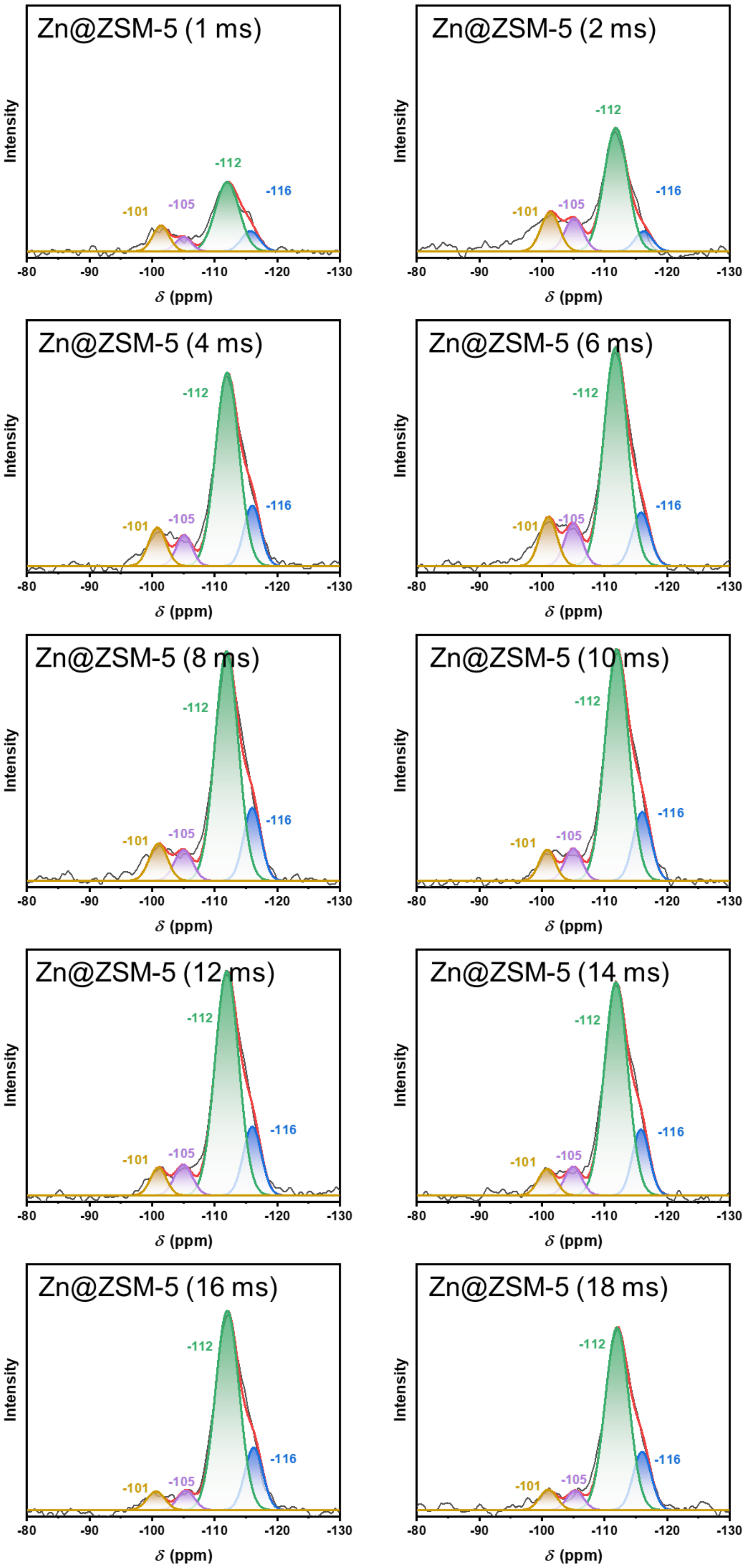

Figure S4. ^{1}H-^{29}Si CP/MAS NMR spectra of Zn@ZSM-5 at different contact time before calcination.

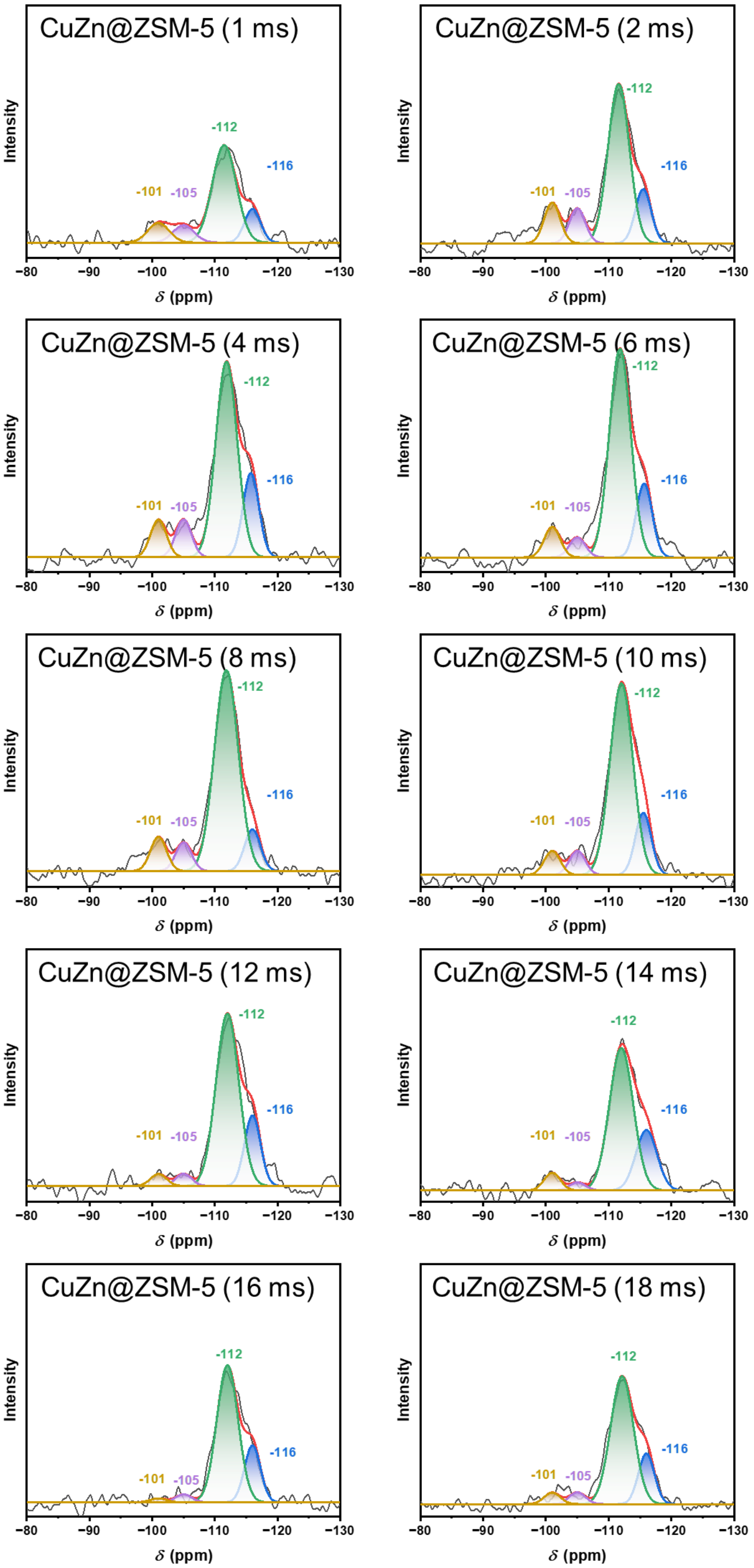

Figure S5. ^{1}H-^{29}Si CP/MAS NMR spectra of CuZn@ZSM-5 at different contact time before calcination.

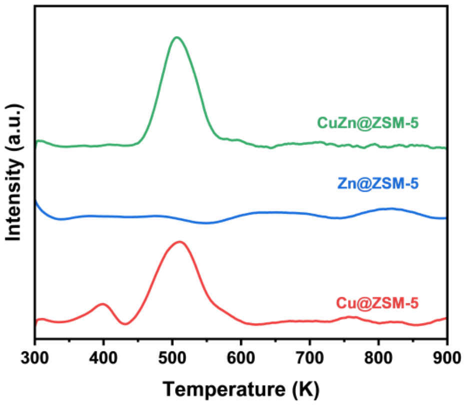

Figure S6. H_2-TPR curves of (a) ZSM-5, (b) Cu@ZSM-5, (c) Zn@ZSM-5 and (d) CuZn@ZSM-5.

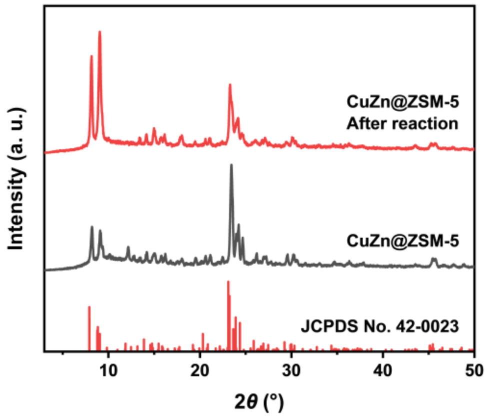

Figure S7. XRD patterns of CuZn@ZSM-5 catalyst before and after 50 h reaction.

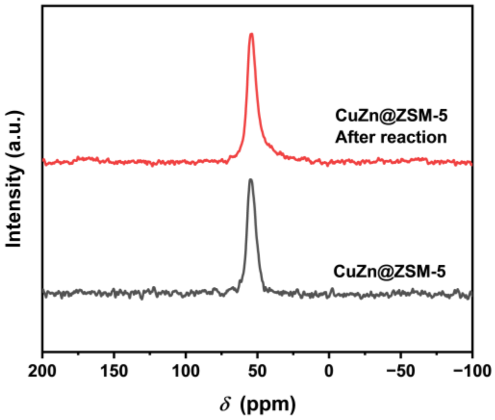

Figure S8. ^{27}Al MAS NMR spectra of CuZn@ZSM-5 catalyst before and after 50 h reaction.

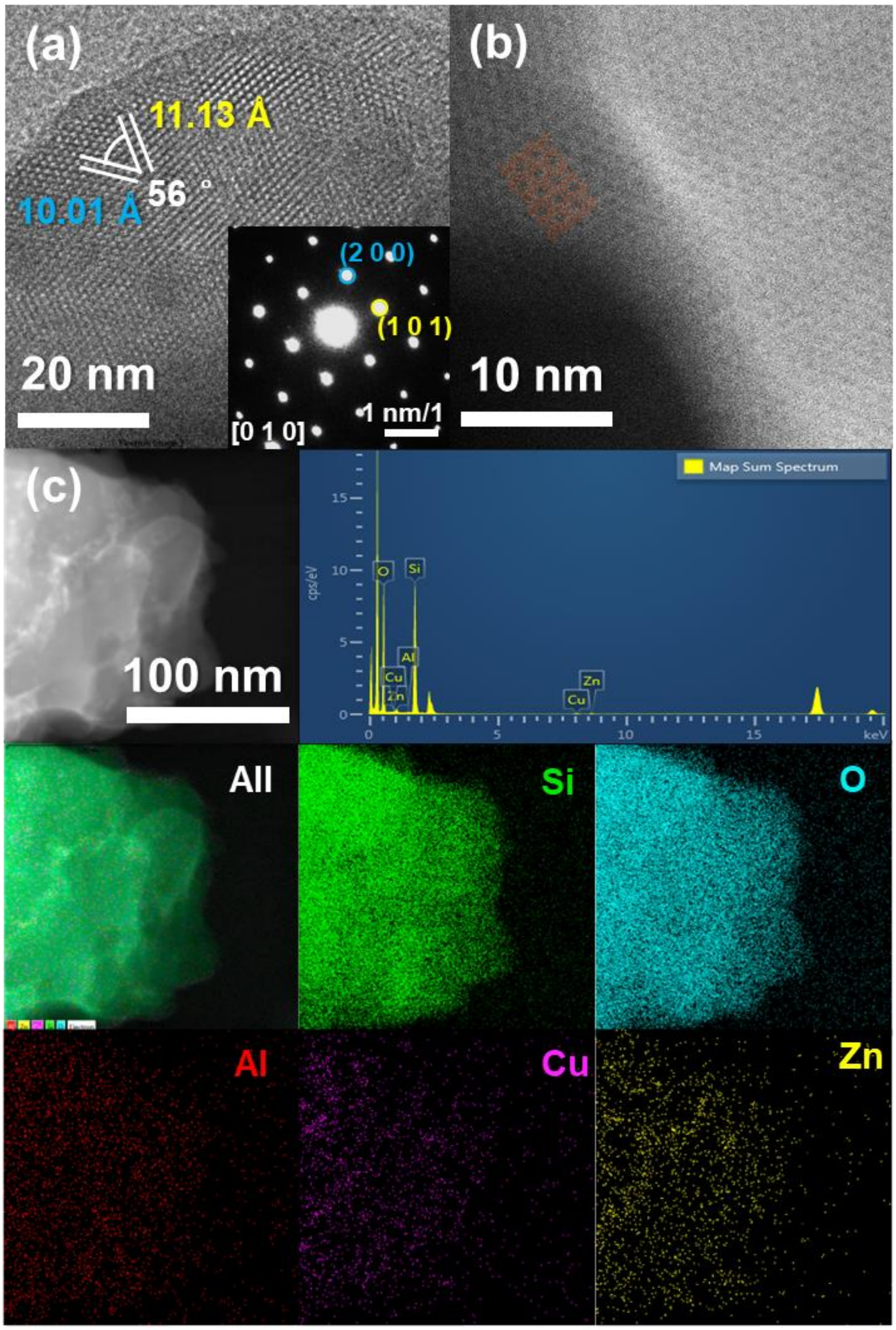

Figure S9. (a) HRTEM and SAED (inset), (b) HAADF-STEM and (c) EDS of CuZn@ZSM-5 after 50 h reaction.

Table S1. Crystallographic and TOPAS fitting parameters.

Sample	Cu@ZSM-5	Zn@ZSM-5	CuZn@ZSM-5
Crystal system	Monoclinic	Monoclinic	Monoclinic
Space group	*P121/n1*	*P121/n1*	*P121/n1*
Composition	$Al_{1.20}Si_{94.80}O_{192}$	$Al_{1.16}Si_{94.84}O_{192}$	$Al_{1.26}Si_{94.74}O_{192}$
a / Å	19.89136(1)	19.89116(2)	19.89531(3)
b / Å	20.11609(3)	20.11212(6)	20.10733(8)
c / Å	13.38432(2)	13.37848(3)	13.37835(6)
α / °	90.0000	90.0000	90.0000
β / °	90.5726 (9)	90.5966(8)	90.5713(3)
γ / °	90.0000	90.0000	90.0000
*R_{wp} / %	7.59	9.23	9.69
Wavelength / Å	0.68860 (6)	0.68860 (6)	0.68860 (6)
2θ range / °	2 - 40	2 - 40	2 - 40

* R_{wp}: $w = w'[1 - (||F_0| - |F_c||/6\sigma(F_0))^2]^2$, while $w' = 1/\Sigma_r A_r T_r(X)$, X is F_c/F_c(max).

Table S2. Atomic information from the Rietveld refinement of Cu@ZSM-5.

Atom	*x*	*y*	*z*	SOF	B_{eq}
Si1	0.05546	0.42056	-0.3199	1	0.26
Si2	0.0309	0.31368	-0.16358	1	0.29
Si3	0.06249	0.2796	0.05346	1	0.29
Si4	0.06233	0.12395	0.03674	1	0.29
Si5	0.02804	0.07678	-0.15797	1	0.26
Si6	0.05585	0.19556	-0.31331	1	0.26
Si7	-0.17148	0.42542	-0.3193	1	0.29
Si8	-0.12653	0.31225	-0.17388	1	0.29
Si9	-0.1759	0.27325	0.03597	1	0.26
Si10	-0.17634	0.11895	0.03436	1	0.29
Si11	-0.12939	0.07156	-0.17516	1	0.29
Si12	-0.16528	0.19079	-0.31408	1	0.29
Si13	0.44297	0.42837	-0.33456	1	0.26
Si14	0.47305	0.31237	-0.18814	1	0.29
Si15	0.43889	0.27704	0.0294	1	0.29
Si16	0.43563	0.12155	0.0338	1	0.29
Si17	0.47315	0.07096	-0.17844	1	0.26
Si18	0.43778	0.18737	-0.31743	1	0.29
Si19	0.67204	0.42389	-0.31417	1	0.26
Si20	0.6313	0.31278	-0.16836	1	0.32
Si21	0.66892	0.27312	0.04608	1	0.29
Si22	0.67007	0.11867	0.03868	1	0.29
Si23	0.63082	0.07268	-0.17757	1	0.32
Si24	0.6807	0.19446	-0.29789	1	0.26
O1	0.0588	0.3779	-0.2194	1	0.79
O2	0.0662	0.3106	-0.0564	1	0.63
O3	0.0472	0.2018	0.0465	1	0.92
O4	0.0671	0.1032	-0.0784	1	0.63
O5	0.0443	0.123	-0.2693	1	0.66
O6	0.0477	0.2483	-0.2248	1	0.89
O7	-0.1533	0.3769	-0.2289	1	0.89
O8	-0.1669	0.305	-0.0725	1	0.79
O9	-0.1558	0.196	0.0316	1	0.76
O10	-0.1689	0.0885	-0.0753	1	0.89
O11	-0.1511	0.1208	-0.263	1	0.87
O12	-0.1376	0.2483	-0.2424	1	0.95
O13	-0.0485	0.3189	-0.149	1	1.03
O14	-0.0509	0.0781	-0.1529	1	0.74
O15	0.1253	0.4145	-0.3771	1	0.82
O16	-0.0041	0.3923	-0.3892	1	0.87
O17	-0.134	0.4022	-0.4186	1	0.71
O18	0.1298	0.2003	-0.3583	1	0.63

O19	0.0026	0.2099	-0.4008	1	0.82
O20	-0.1275	0.1948	-0.4188	1	0.68
O21	0.0515	0.0032	-0.2041	1	0.58
O22	-0.1475	-0.0023	-0.2098	1	0.68
O23	-0.2501	0.4239	-0.3413	1	0.82
O24	-0.2435	0.1987	-0.3356	1	0.68
O25	-0.2525	0.2822	0.0676	1	0.61
O26	-0.2526	0.1101	0.0697	1	0.53
O27	0.4503	0.3799	-0.2408	1	0.92
O28	0.448	0.3143	-0.0754	1	0.68
O29	0.4318	0.1991	0.0094	1	0.82
O30	0.4478	0.0812	-0.0669	1	0.84
O31	0.4351	0.1206	-0.2527	1	0.63
O32	0.4401	0.2505	-0.2451	1	0.79
O33	0.659	0.3797	-0.2169	1	0.74
O34	0.6459	0.3148	-0.0508	1	0.58
O35	0.6513	0.1961	0.027	1	0.68
O36	0.6559	0.0822	-0.0653	1	0.92
O37	0.6678	0.1232	-0.2504	1	0.92
O38	0.6694	0.2497	-0.2144	1	0.84
O39	0.553	0.3054	-0.1913	1	1.05
O40	0.5519	0.0851	-0.1834	1	0.84
O41	0.3714	0.4186	-0.3885	1	0.89
O42	0.5015	0.4154	-0.4135	1	0.76
O43	0.632	0.3938	-0.4087	1	0.74
O44	0.3711	0.19	-0.3847	1	0.66
O45	0.5032	0.1862	-0.3863	1	0.74
O46	0.6326	0.2074	-0.3914	1	0.92
O47	0.4576	-0.0039	-0.2104	1	0.66
O48	0.6481	-0.0013	-0.212	1	0.71
Cu1	0.64183	-0.00317	-0.5223	0.23388*	0.71
Cu2	0.74832	0.21402	-0.65127	0.19472*	0.71

*Actual Cu content according to ICP-AES results.

Table S3. Atomic information from the Rietveld refinement of Zn@ZSM-5.

Atom	*x*	*y*	*z*	SOF	B_{eq}
Si1	0.0656	0.41667	-0.322	1	0.26
Si2	0.02528	0.31504	-0.16531	1	0.29
Si3	0.06062	0.27932	0.05372	1	0.29
Si4	0.06589	0.12686	0.03694	1	0.29
Si5	0.0257	0.07348	-0.17146	1	0.26
Si6	0.05499	0.19668	-0.31442	1	0.26
Si7	-0.16663	0.43089	-0.32012	1	0.29
Si8	-0.12374	0.31296	-0.17935	1	0.29
Si9	-0.16941	0.27205	0.03669	1	0.26
Si10	-0.17416	0.11869	0.03951	1	0.29
Si11	-0.11563	0.06764	-0.17407	1	0.29
Si12	-0.16817	0.18949	-0.32317	1	0.29
Si13	0.43731	0.42989	-0.33947	1	0.26
Si14	0.46939	0.31332	-0.18952	1	0.29
Si15	0.44655	0.27606	0.033	1	0.29
Si16	0.42496	0.12016	0.03929	1	0.29
Si17	0.47259	0.07461	-0.17748	1	0.26
Si18	0.43863	0.18243	-0.3174	1	0.29
Si19	0.66773	0.42281	-0.31661	1	0.26
Si20	0.63264	0.31436	-0.1662	1	0.32
Si21	0.6685	0.27473	0.04518	1	0.29
Si22	0.66797	0.11392	0.04027	1	0.29
Si23	0.62854	0.06969	-0.17729	1	0.32
Si24	0.68495	0.19307	-0.2972	1	0.26
O1	0.05518	0.37772	-0.22374	1	0.79
O2	0.06838	0.31645	-0.05194	1	0.63
O3	0.05523	0.20425	0.02875	1	0.92
O4	0.06336	0.11374	-0.06722	1	0.63
O5	0.03216	0.12576	-0.25003	1	0.66
O6	0.06271	0.24792	-0.23497	1	0.89
O7	-0.14127	0.37654	-0.2228	1	0.89
O8	-0.15847	0.31028	-0.05787	1	0.79
O9	-0.15706	0.19975	0.03376	1	0.76
O10	-0.16391	0.0822	-0.06986	1	0.89
O11	-0.15729	0.13041	-0.26722	1	0.87
O12	-0.15422	0.24054	-0.25511	1	0.95
O13	-0.0576	0.32383	-0.14702	1	1.03
O14	-0.04198	0.07707	-0.15548	1	0.74
O15	0.13342	0.42032	-0.38828	1	0.82
O16	0.01107	0.38923	-0.39571	1	0.87
O17	-0.13513	0.40291	-0.43132	1	0.71
O18	0.13214	0.20946	-0.35605	1	0.63

O19	0.01387	0.20358	-0.40424	1	0.82
O20	-0.1337	0.20542	-0.41264	1	0.68
O21	0.03681	0.00131	-0.2034	1	0.58
O22	-0.15655	-0.00251	-0.21037	1	0.68
O23	-0.26224	0.41359	-0.34105	1	0.82
O24	-0.237	0.18828	-0.31545	1	0.68
O25	-0.24882	0.27485	0.06576	1	0.61
O26	-0.25156	0.10784	0.06164	1	0.53
O27	0.43823	0.3811	-0.24549	1	0.92
O28	0.45308	0.30404	-0.08033	1	0.68
O29	0.42042	0.20095	0.00261	1	0.82
O30	0.43792	0.08485	-0.08596	1	0.84
O31	0.44419	0.11432	-0.25987	1	0.63
O32	0.42742	0.25079	-0.23057	1	0.79
O33	0.65959	0.37988	-0.22073	1	0.74
O34	0.65498	0.30448	-0.05834	1	0.58
O35	0.66363	0.20385	0.03821	1	0.68
O36	0.65409	0.07585	-0.05784	1	0.92
O37	0.66256	0.12362	-0.24572	1	0.92
O38	0.6681	0.25589	-0.20484	1	0.84
O39	0.55151	0.3075	-0.16607	1	1.05
O40	0.54524	0.08374	-0.20822	1	0.84
O41	0.36138	0.41686	-0.38434	1	0.89
O42	0.49194	0.40929	-0.4009	1	0.76
O43	0.64761	0.3908	-0.41325	1	0.74
O44	0.36516	0.1973	-0.38527	1	0.66
O45	0.5104	0.18921	-0.39782	1	0.74
O46	0.62145	0.20459	-0.38865	1	0.92
O47	0.4531	0.00028	-0.19418	1	0.66
O48	0.64266	0.00514	-0.21471	1	0.71
Zn	0.7262	0.33496	-0.61267	0.10945*	0.71

*Actual Zn content according to ICP-AES results.

Table S4. Atomic information from the Rietveld refinement of CuZn@ZSM-5.

Atom	*x*	*y*	*z*	SOF	B_{eq}
Si1	0.05546	0.42056	-0.3199	1	0.26
Si2	0.0309	0.31368	-0.16358	1	0.29
Si3	0.06249	0.2796	0.05346	1	0.29
Si4	0.06233	0.12395	0.03674	1	0.29
Si5	0.02804	0.07678	-0.15797	1	0.26
Si6	0.05585	0.19556	-0.31331	1	0.26
Si7	-0.17148	0.42542	-0.3193	1	0.29
Si8	-0.12653	0.31225	-0.17388	1	0.29
Si9	-0.1759	0.27325	0.03597	1	0.26
Si10	-0.17634	0.11895	0.03436	1	0.29
Si11	-0.12939	0.07156	-0.17516	1	0.29
Si12	-0.16528	0.19079	-0.31408	1	0.29
Si13	0.44297	0.42837	-0.33456	1	0.26
Si14	0.47305	0.31237	-0.18814	1	0.29
Si15	0.43889	0.27704	0.0294	1	0.29
Si16	0.43563	0.12155	0.0338	1	0.29
Si17	0.47315	0.07096	-0.17844	1	0.26
Si18	0.43778	0.18737	-0.31743	1	0.29
Si19	0.67204	0.42389	-0.31417	1	0.26
Si20	0.6313	0.31278	-0.16836	1	0.32
Si21	0.66892	0.27312	0.04608	1	0.29
Si22	0.67007	0.11867	0.03868	1	0.29
Si23	0.63082	0.07268	-0.17757	1	0.32
Si24	0.6807	0.19446	-0.29789	1	0.26
O1	0.0588	0.3779	-0.2194	1	0.79
O2	0.0662	0.3106	-0.0564	1	0.63
O3	0.0472	0.2018	0.0465	1	0.92
O4	0.0671	0.1032	-0.0784	1	0.63
O5	0.0443	0.123	-0.2693	1	0.66
O6	0.0477	0.2483	-0.2248	1	0.89
O7	-0.1533	0.3769	-0.2289	1	0.89
O8	-0.1669	0.305	-0.0725	1	0.79
O9	-0.1558	0.196	0.0316	1	0.76
O10	-0.1689	0.0885	-0.0753	1	0.89
O11	-0.1511	0.1208	-0.263	1	0.87
O12	-0.1376	0.2483	-0.2424	1	0.95
O13	-0.0485	0.3189	-0.149	1	1.03
O14	-0.0509	0.0781	-0.1529	1	0.74
O15	0.1253	0.4145	-0.3771	1	0.82
O16	-0.0041	0.3923	-0.3892	1	0.87
O17	-0.134	0.4022	-0.4186	1	0.71
O18	0.1298	0.2003	-0.3583	1	0.63

O19	0.0026	0.2099	-0.4008	1	0.82
O20	-0.1275	0.1948	-0.4188	1	0.68
O21	0.0515	0.0032	-0.2041	1	0.58
O22	-0.1475	-0.0023	-0.2098	1	0.68
O23	-0.2501	0.4239	-0.3413	1	0.82
O24	-0.2435	0.1987	-0.3356	1	0.68
O25	-0.2525	0.2822	0.0676	1	0.61
O26	-0.2526	0.1101	0.0697	1	0.53
O27	0.4503	0.3799	-0.2408	1	0.92
O28	0.448	0.3143	-0.0754	1	0.68
O29	0.4318	0.1991	0.0094	1	0.82
O30	0.4478	0.0812	-0.0669	1	0.84
O31	0.4351	0.1206	-0.2527	1	0.63
O32	0.4401	0.2505	-0.2451	1	0.79
O33	0.659	0.3797	-0.2169	1	0.74
O34	0.6459	0.3148	-0.0508	1	0.58
O35	0.6513	0.1961	0.027	1	0.68
O36	0.6559	0.0822	-0.0653	1	0.92
O37	0.6678	0.1232	-0.2504	1	0.92
O38	0.6694	0.2497	-0.2144	1	0.84
O39	0.553	0.3054	-0.1913	1	1.05
O40	0.5519	0.0851	-0.1834	1	0.84
O41	0.3714	0.4186	-0.3885	1	0.89
O42	0.5015	0.4154	-0.4135	1	0.76
O43	0.632	0.3938	-0.4087	1	0.74
O44	0.3711	0.19	-0.3847	1	0.66
O45	0.5032	0.1862	-0.3863	1	0.74
O46	0.6326	0.2074	-0.3914	1	0.92
O47	0.4576	-0.0039	-0.2104	1	0.66
O48	0.6481	-0.0013	-0.212	1	0.71
Cu1	0.77813	-0.09001	-0.81257	0.20542*	0.71
Cu2	0.76327	0.09295	-0.61284	0.14961*	0.71
Zn	0.96102	-0.00289	-0.52892	0.14587*	0.71

*Actual Cu and Zn content according to ICP-AES results.

Table S5. Binding energy of Cu $2p_{3/2}$ and Zn $2p_{3/2}$.

Compound	$2p_{3/2}$ Binding Energy (eV)	Reference
Cu	932.6	Table 8 in Ref. [48]
Cu^{+}	932.2	Table 8 in Ref. [48]
Cu^{2+}	933.7	Table 8 in Ref. [48]
Cu^{2+}	934.7	Table 8 in Ref. [48]
Cu^{2+}(satellite peak)	944.1	Table 8 in Ref. [48]
Cu^{2+}	933.7	This work

Cu^{2+}	935.3	This work
Cu^{2+}(satellite peak)	945.3	This work
Zn	1021.6	Table 1 in in Ref. [49]
Zn^{2+}	1022.4	Table 1 in in Ref. [49]
